# Breastfeeding practices in urban and rural Vietnam

**DOI:** 10.1186/1471-2458-12-964

**Published:** 2012-11-12

**Authors:** Huong Nguyen Thu, Bo Eriksson, Toan Tran Khanh, Max Petzold, Göran Bondjers, Chuc Nguyen Thi Kim, Liem Nguyen Thanh, Henry Ascher

**Affiliations:** 1 Research Institute for Child Health, National Hospital of Pediatrics, 18/879 La Thanh road, Dong Da district, Hanoi, Vietnam; 2 Nordic School of Public Health, PO Box 12133, SE-402 42, Gothenburg, Sweden; 3 Family Medicine Department, Hanoi Medical University, No.1 Ton That Tung Street, Hanoi, Vietnam; 4 Sahlgrenska Academy, University of Gothenburg, PO Box 440, SE-405 30, Gothenburg, Sweden

**Keywords:** Breastfeeding, Urban, Rural, Hanoi, Vietnam

## Abstract

**Background:**

The aim of this study was to describe and compare breastfeeding practices in rural and urban areas of Vietnam and to study associations with possibly influencing person and household factors. This type of study has not been conducted in Vietnam before.

**Methods:**

Totally 2,690 children, born from 1st March 2008 to 30th June 2010 in one rural and one urban Health and Demographic Surveillance Site, were followed from birth to the age of 12 months. Information about demography, economy and education for persons and households was obtained from household surveys. Standard statistical methods including survival and regression analyses were used.

**Results:**

Initiation of breastfeeding during the first hour of life was more frequent in the urban area compared to the rural (boys 40% vs. 35%, girls 49% vs. 40%). High birth weight and living in households with large number of assets significantly increased the probability for early initiation of breastfeeding. Exclusive breastfeeding at three months of age was more commonly reported in the rural than in the urban area (boys 58% vs. 46%, girls 65% vs. 53%). The duration of exclusive breastfeeding as well as of any breastfeeding was longer in the rural area than in the urban area (medians for boys 97 days vs. 81 days, for girls 102 days vs. 91 days). The percentages of children with exclusive breastfeeding lasting at least 6 months, as recommended by WHO, were low in both areas. The duration of exclusive breastfeeding was significantly shorter for mothers with three or more antenatal care visits or Caesarean section in both areas. High education level of mothers was associated with longer duration of exclusive breastfeeding in the rural area. No significant associations were found between duration of exclusive breastfeeding and mother’s age, household economy indicators or household size.

**Conclusion:**

Intervention programs with the aim to promote breastfeeding are needed. Mothers should particularly be informed about the importance of starting breastfeeding early and to prolong exclusive breastfeeding. In order to reach the WHO recommendation of six months exclusive breastfeeding, we propose an extended maternity leave legislation to at least six months.

## Background

The benefits of breastfeeding for infants, mothers and communities are widely acknowledged [1]. Breastfeeding can decrease the incidence and severity of infectious diseases and thereby the risk for post neonatal infant death [1]. Exclusive breastfeeding has been found to prevent obesity [1]. Breastfeeding has also been found to be associated with better cognitive development [1] as well as reduced occurrence of postpartum bleedings, breast cancer and ovarian cancer of mothers [2]. In addition, benefits of breastfeeding on the economic situation of the family and the environment have been reported [1]. The World Health Organization (WHO) recommends exclusive breastfeeding for the first 6 months of life, with continued breastfeeding through the second year of life [3]. However, these recommendations are not followed everywhere: in 2007, a national survey in USA reported that 26% of all women, with children aged from 0 to 5 years, did not give any breastfeeding at all [2]. In a study of five Asian countries 2002 to 2005, the percentages of exclusive breastfeeding of infants younger than 6 months were reported as 30.7% in Timor-Leste, 33.7% in Philippine, 38.9% in Indonesia and 60.1% in Cambodia. The fifth country, Vietnam, reported only 15.5% [3].

In Vietnam breastfeeding has been promoted by health authorities since the early 1980s. The government then adopted a policy for exclusive breastfeeding to at least six months of age as part of the national strategy and advocated initiation of breastfeeding during the first hour after birth [4].

In practice the recommendations have not been followed [3]. A study from Ho Chi Minh city in 2000 reported that only 47.5% of all newborns were given breast milk as the first feed [5]. In a rural area in 2002, initiation of breastfeeding during the first hour of life was reported for 73.6% of all newborn and 83.6% were exclusively breastfed at one week of age [6]. However, exclusive breastfeeding dropped to zero by 6 months [7]. In another study 77% and 22% of children continued any breastfeeding at 12 months and 24 months of age respectively [8].

In Vietnam, it is generally poorly understood by health professionals what factors are associated with breastfeeding practices [9]. Cultural and traditional beliefs, socioeconomic situation [10], mother’s work outside home [11], mother’s educational level, father’s occupation [7], mother’s lack of knowledge and misinformation [12], the delivery method and delivery location as well as health problems of the mother [6] have been proposed to influence the breast feeding practices. Another potential factor of influence is the maternity leave regulation. In Vietnam the maternity leave is at present 4 months [13].

Rural–urban differences in breastfeeding initiation, exclusive breastfeeding, and duration of breastfeeding have been reported from some countries [2,14-17]. The prevalence of breastfeeding was higher in an urban setting as compared to a rural in USA and Tanzania [2,17]. The opposite was found in China [14].

Longitudinal studies like the present that compare breastfeeding practices between urban and rural areas have not been conducted in Vietnam. A hypothesis is that there are different breastfeeding practices of infants in rural and urban areas in Vietnam, possibly due to differences in economic resources and the educational level of mothers. The aim of this study is therefore to quantitatively describe breastfeeding practices during the first year of life in one rural and one urban area of Vietnam using information from repeated interviews with mothers and to study associations between child, mother and household factors and these breastfeeding practices.

## Methods

### Study sites

The study was conducted in two Health and Demographic Surveillance Sites (HDSS), one urban and one rural, located within the municipality of Hanoi, the capital city of Vietnam. Dong Da is a central, old district of urban Hanoi. The total population is about 352,000 persons living in 21 communes with a total area of around 10 km^2^. The socio-economic characteristics are relatively typical for urban Vietnamese areas in big cities with an estimated income per capita above 1,000 USD per year. Three communes with 37,000 persons in 10,500 households strategically selected to have different economic levels, were defined as the DodaLab HDSS [18].

Ba Vi is a rural district within the municipality of Hanoi with a population of 250,000 persons. Farming is the main occupation with an income per capita of about 300 USD per year. A sample called FilaBavi HDSS, of 11,089 households with 51,024 persons has been followed since 1999 [19].

A cross-sectional baseline study was undertaken in DodaLab from the end of 2007 and into the beginning of 2008 to obtain information about persons and households, mainly on demographic characteristics, education and occupation as well as the social and economic household situation. Corresponding surveys are conducted every second year in FilaBavi, the two latest in 2007 and 2009. Information from the 2007 surveys in both sites has been used for this study. At both sites, all households are routinely visited every three months to record vital events, births, deaths and migrations. Every year, about 500 children are born in DodaLab and about 900 are born yearly in FilaBavi. The quarterly surveys have been used to identify pregnant women for the present study but the subsequent follow up of pregnant women and their children have then been conducted separately.

### Study design and subjects

All mothers of infants born from 1^st^ March, 2008 to 30^th^ June 2010, were invited to participate in the study. All had taken part in a previous study of antenatal and delivery care [18]. The infants included were then followed from birth to one year of age with respect to breastfeeding. Information about breastfeeding and exclusive breastfeeding was collected in monthly interviews. Totally 2,690 babies were enrolled for the study. During the first months of the data collection, some observations in FilaBavi were missed due to mistakes during the field work. For 57 infants no breastfeeding data was received resulting in 2,633 remaining for the study (1,145 in DodaLab, 633 boys and 512 girls and 1,488 in FilaBavi, 823 boys and 665 girls). The total numbers of breastfeeding interviews were 10,189 in DodaLab and 17,008 in FilaBavi.

### Data collection

Information on the infant feeding was obtained through interviews with the mothers using a structured questionnaire constructed by the investigators with references to Scott JA [20] and Dhandapany G et al. [21]. The data collection was approved by the Research Committee of Hanoi Medical University. The questionnaire was pretested for cultural sensitivity before the actual data collection. The mothers were interviewed each month from delivery to child age of twelve months. At each interview the mothers were asked if the baby had received only breastfeeding or breastfeeding together with additional food or no breastfeeding at all during the period since the previous interview. Data was collected by 106 HDSS interviewers (46 in FilaBavi and 60 in DodaLab), who were trained on household interview skills and the content of the questionnaire. The interviewers were trained to carefully ask about the feeding details of the infants during the period since the last interview, or birth in the first interview. As a quality control 3% of the randomly selected forms for re-interview by field supervisors. One hundred percent of the forms were rechecked by field supervisors and data clerks before entering into computers.

### WHO breastfeeding definitions

To describe breastfeeding, the World Health Organisation provides the following definitions [22]:

Exclusive breastfeeding: The infant receives breast milk, from the breast of the mother or a wet nurse or expressed, with the only additional oral intake of oral rehydration solutions (ORS) or medication including vitamins or minerals.

*Any breastfeeding: The infant receives breast milk, from the breast of the mother or a wet nurse or expressed, with or without additional oral foods. This category includes the WHO definitions of exclusive breastfeeding as well as non-exclusive breastfeeding, that is predominant breastfeeding and complementary feeding according to the WHO definitions*.

Given the way data has been obtained for this article we are not able to discuss exclusive breastfeeding in a strict sense. Even if a mother by the first interview report that the child has not been given anything but breast milk we can’t be sure that prelacteal feeds have not been given. Early single meals of formula given by the maternity ward staff cannot be fully excluded. The importance of such deviations from exclusive breastfeeding is different in different situations. e.g. in a study of exclusive breastfeeding and allergy development it can be extremely important to know about prelacteal feeds. For studies of child growth the importance could be considered minute.

For the aim of this paper we wanted to study three components. The first is the frequency of early onset of breastfeeding defined as within one hour after delivery. The second is the duration for which mothers report that the infant is only given breast milk (Exclusive breastfeeding). The third is the duration for which the infant is reported to be given breast milk with or without additional food (Any breastfeeding) If a visit with report of exclusive breastfeeding was followed by a visit reporting any supplementary food or no breastfeeding, but exclusive breastfeeding was reported at later visits, the end time of exclusive breastfeeding was defined by the first interruption.

### Statistical analysis

#### Outcomes

As described above, two time variables measuring breastfeeding time were defined, (i) the number of days from birth to the maximum time with exclusive breastfeeding reported and (ii) the number of days from birth to the maximum time with any breastfeeding reported. A dichotomous outcome variable “early commencement of breastfeeding” was defined as initiation of breastfeeding in the first hour after delivery.

#### Explanatory variables

At the child level we included sex of the child, birth weight, delivery in hospital and in particular use of Caesarean section as explanatory variables.

For mothers, the following variables were used: age, education, occupation and number of antenatal care visits. Parity was not included as it is strongly correlated with age creating risk for collinearity problems in the estimation. Also, parity is less informative since it is commonly only zero (nullipara) or one due to the two-child policy. Education and occupation are also correlated but for breastfeeding we should expect occupation to have some importance *per se,* predominantly farming in the rural area and office employment in the urban.

The durations of exclusive breastfeeding or any breastfeeding were described using Kaplan Meier curves and analysed by means of standard survival analysis technique including stratification, regression and Cox regression models. In the analysis of exclusive breastfeeding, infants never reported to have been exclusively breastfed were not included and infants never reported to have been breastfed were not included in the analysis of any breastfeeding.

Two variables were used for stratification throughout the analysis: context, i.e. living in urban or rural area, and sex of the child. We have previously found that these variables define natural strata with respect to birth weight and growth during the first year of life [23]. Cox regressions of breastfeeding duration were conducted separately within each stratum. In addition specific studies of medians of exclusive breastfeeding were done for selected variables. Associations between the outcome variable “early initiation” and the explanatory variables were investigated using multiple logistic regressions. Statements of statistical significance have p<0.05 as the default level.

### Ethical consideration

Approval for the project was given by the Scientific and Ethical Committee of Hanoi Medical University, the Hanoi Health Bureau and the Dong Da district authorities. The proposal was also approved and given permission by Ministry of Health after the baseline survey.

The participants were informed about the purpose of the studies and their right to decline participation or to withdraw at any time and without any conditions. Consent was sought from all eligible mothers and those who consented were included in the study. Data were analysed and results were presented anonymously. All results were duly disseminated to communities and authorities.

## Results

Early initiation of breastfeeding during the first hour was significantly more common in urban as compared to rural newborns (Table 1). The differences between sites as well as between the sexes were statistically significant (p< 0.01). A logistic regression using all listed independent variables showed that high birth weight significantly increased the probability for early initiation (p<0.001). The use of Caesarean section on the other hand delayed initiation. The odds ratio for early initiation was 0.12 with p<0.001 comparing children delivered with Caesarean section to other. Increasing number of household assets showed a tendency towards increasing probability of early initiation though not statistically significant (p=0.068).

**Table 1:** Initiation of breastfeeding by site and child sex

	**Urban boys**	**Urban girls**	**Rural boys**	**Rural girls**
Total number of children	633	512	823	665
Number of children with breastfeeding initiated during first hour	254 (40%)	251 (49%)	291 (35%)	270 (40%)
Number of children with breastfeeding initiated during first 24 hours	272 (43%)	351 (68.5%)	414 (50.3%)	328 (49.3%)

**Figure 1:**
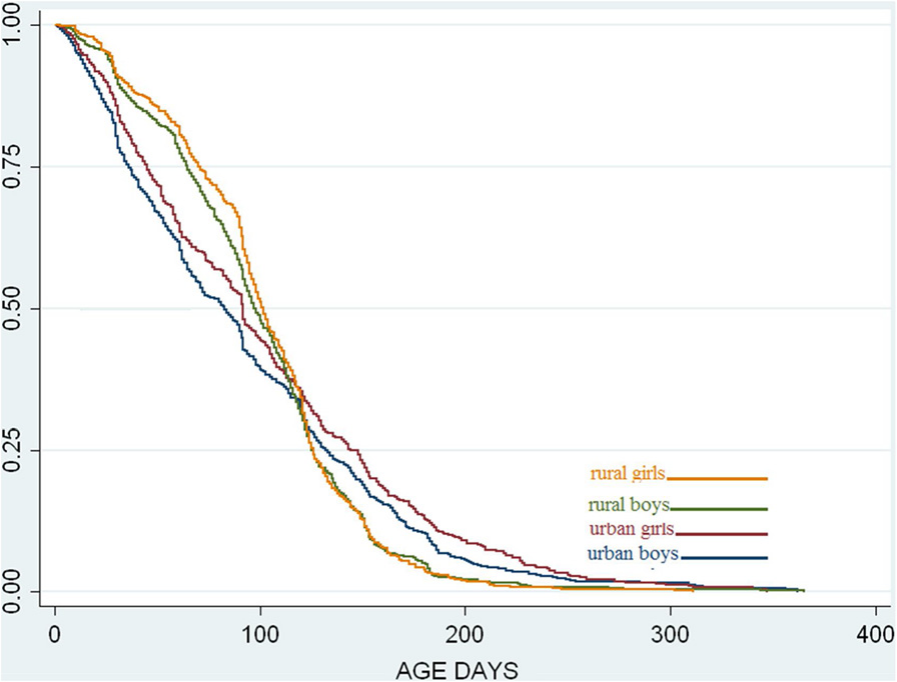
**Estimated proportions of exclusive breastfeeding as a function of age for urban and rural infants.** Children never reporting exclusive breastfeeding are not included.

Exclusive breastfeeding in the first months was significantly more common in the rural area than in the urban (Figure 1, Table 2). The differences at age three months were 12 percent units, both for boys and girls. A reverse relation was observed at a higher age, however based on observations from fewer children and therefore more likely to be a matter of chance. Also in a corresponding figure based only on children with 9–12 visits this difference became weaker and not statistically significant. At all ages and at both sites girls were more frequently exclusively breastfed but the differences were not statistically significant. The differences in medians of the duration of exclusive breastfeeding between areas were statistically significant (p < 0.001) as well as sexes (p = 0.016) (Table 2).

**Table 2:** Exclusive breastfeeding at selected ages and duration of exclusive breastfeeding by site and child sex

	**Urban boys**	**Urban girls**	**Rural boys**	**Rural girls**
Exclusive breastfeeding at 1 month (%) 95% confidence interval	81 (78;84)	86 (83;89)	91 (88;94)	91 (89;93)
Exclusive breastfeeding at 3 month (%) 95% confidence interval	46 (42;50)	53 (49;58)	58 (53;60)	65 (60;68)
Exclusive breastfeeding at 6 months (%) 95% confidence interval	8 (4;12)	11 (7;15)	4 (0;9)	4 (0;9)
Medians of duration of exclusive breastfeeding with 95% confidence interval (days)	81 (69;89)	91 (84;95)	97 (94;102)	102 (98;105)

**Figure 2:**
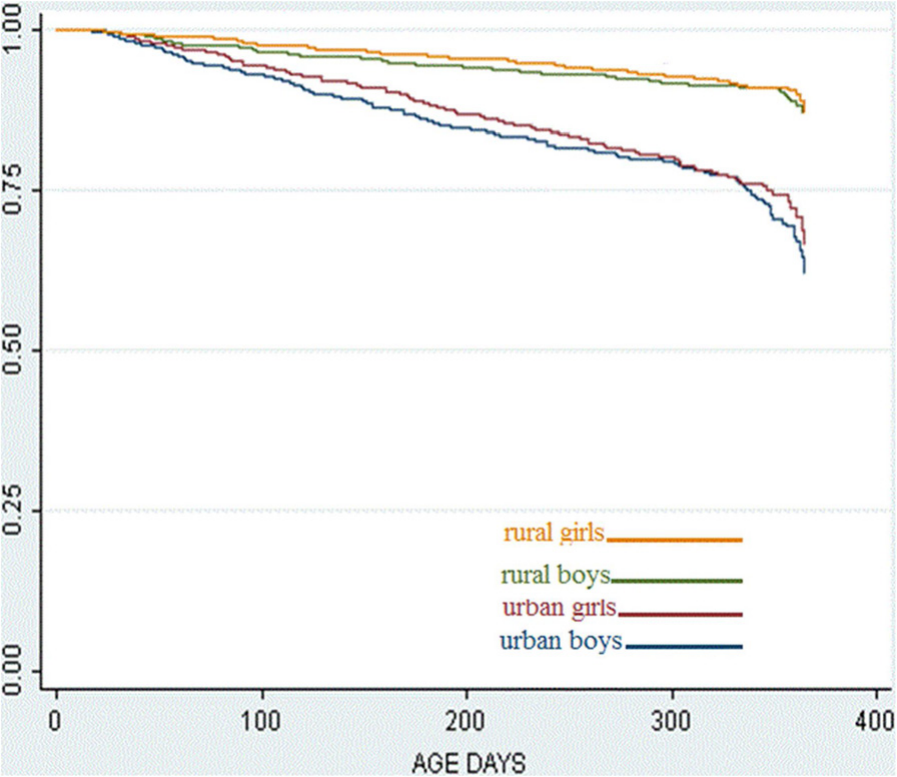
**Estimated proportions of any breastfeeding as a function of age for urban and rural infants.** Children never reporting any breastfeeding are not included.

Most infants, in both areas, were given any breastfeeding for the first 6–9 month. Later, breastfeeding declined, most rapidly in the urban area (Figure 2).

In the urban area a trend to shorter exclusive breastfeeding for mothers with longer education was seen. The median durations were 108 days for mothers with secondary school, 88 days for high school and 82 days for higher education. This trend was however, not statistically significant. In the rural area the trend was opposite with longer breastfeeding in mothers with longer education; 98, 98 and 108 days, respectively. Here the difference was statistically significant.

Mothers reporting farming as occupation in the rural area and being employed in offices and business in the urban area did not differ from others with respect to exclusive breastfeeding duration medians. Likewise no statistically significant associations or systematic patterns were found between exclusive breastfeeding duration and mother’s age, household economy indicators or household size.

The median exclusive breastfeeding duration for those attending less than three antenatal care visits was 105 days, whereas the duration for those attending three visits or more was significantly shorter, 92 days (p<0.05). The majority of urban infants were born in hospitals. Those infants had shorter duration of exclusive breastfeeding than the urban infants born outside the hospital (medians are 85 days and 129 days). In the rural area, the opposite trend was found (medians of 113 and 98 days). Both differences were however, non-significant. For children delivered by Caesarean section the median duration was 78 days. The difference was statistically significant from the 98 days for normal deliveries with the same tendency for both sites.

The results of the Cox regressions of exclusive breastfeeding duration on all independent variables within the four strata formed by child sex and area type largely confirmed the association between duration of exclusive breastfeeding and use of Caesarean section. The hazard ratio (HR) for ending exclusive breastfeeding was estimated to 1.25 in the urban and 1.07 in the rural area. High household income turned out to be significantly associated with a HR above one in three out of the four strata. For urban boys, the mother’s age and education were significantly associated with the risk of ending exclusive breastfeeding. Older mothers showed lower risk whereas better educated women exhibited increased risk compared to the overall risk.

The results for median duration of exclusive breastfeeding and the results of Cox regression mainly coincide. The approaches are based on different assumptions, different statistical tests are used and statistical significance (p-values) will differ. Strict application of conventional rules for significance might be considered as creating conflicting results but the discrepancies are mainly indications of a rather complex situation.

Looking at infants given any breastfeeding, the frequency at 12 months of age is high, more than two out of three, in all groups (Table 3). The survival curves for any breastfeeding differ significantly between the urban and the rural areas (p<0.001) but not between sexes (p=0.44) (Figure 2).

**Table 3:** Any breastfeeding at 6 and 12 months of age

	**Urban boys**	**Urban girls**	**Rural boys**	**Rural girls**
Total number of children	633	512	823	665
Number and percentage of children who report any breastfeeding	623 (98.5%)	507 (99%)	820 (99.5%)	665 (99.5)
Any breastfeeding at 6 month (%) 95% confidence interval	85 (81;87)	87 (84;90)	94 (92;96)	96 (94;98)
Any breastfeeding at 12 month (%) 95% confidence interval	66 (61;71)	70 (64;75)	89 (86;91)	90 (88;92)

## Discussion

A key finding of this study was that early initiation of breastfeeding was more common in the urban area than in the rural. A similar finding has been reported from Tanzania where the percentage of initiation of breastfeeding during the first hour after birth was estimated to be 82% in an urban area and 52% in a rural. Only 10% of the urban mothers discarded colostrum compared to 43% of the rural mothers [16]. Delayed initiation of breastfeeding and discarding of colostrum has also been found to be very common in a rural area in Bangladesh. Only 12 % the mothers used colostrum for the first feeding of their newborns and a relationship was found between mother knowledge and the practice of giving colostrum [24]. In Vietnam, comparisons between breastfeeding patterns in rural and urban areas have not previously been published. A study of Vietnamese women in Australia suggested that the proportion of early initiation of breastfeeding was low due to mothers having negative views on colostrum. Only 25.7% thought that colostrum was healthier for babies than formula, 64.9% said that it was equally healthy and 40% gave their babies formula milk in the hospital [25]. In Vietnam, the level of education of mothers in urban areas is generally higher than in rural areas. This might indicate that there is a difference in knowledge about colostrum and the value of early breastfeeding. Vietnam began to implement the policy of “Baby-Friendly Hospitals’ in 1995, which is strongly supportive of breastfeeding [5]. In our study, the proportion of urban mothers who delivered in hospitals was higher than in the rural area [26], which could have contributed to a higher level of early breastfeeding in urban areas. Birth weight, number of household assets and Caesarean section were associated with the pattern of early initiation of breastfeeding in the present study. We also knew that mean birth weight was higher in the urban area than in the rural area [23], Caesarean section was more common [26] and household income was higher [23]. The factors associated with initiation of breastfeeding thus differ between the urban and rural areas. This may partly explain the differences we found in the early initiation of breastfeeding.

The percentages of initiation of breastfeeding during the first hour after birth in the present study were much lower in both areas than the 73.6% found in a rural area of Vietnam in 2002 [6] and the 61.7% reported in a cross-sectional study of National Institute of Nutrition in 2010 [8]. This may indicate that the level of early initiation of breastfeeding does not improve.

An important finding of this study is that exclusive breastfeeding during the first three months was more common in the rural area than in the urban one. This is in line with results from China where exclusive breastfeeding was more common in a rural area (61%) than in an urban (38%) [14]. The opposite was reported from Tanzania where the mean duration of exclusive breastfeeding was longer in the urban setting (23 days) than in the rural (9 days) [16].

Some factors can be suggested to be associated with the differences of exclusive breastfeeding between the two sites. The use of Caesarean section as delivery method has been seen to increase the risk for not breastfeeding in Vietnam and China [6,15]. After surgery, the babies are often taken away from the mother. Mothers can also be worried about side effects of medicines like postoperative antibiotics which may pass to their babies through the breast milk [15]. In the present study the median duration of exclusive breastfeeding of children delivered using Caesarean section was significantly shorter than in other groups in both settings. The percentage of women having Caesarian section was substantially higher in the urban area (38.9%) than in rural area (12.2%) [26]. Caesarian section could be one of the factors behind the differences found in exclusive breastfeeding between the two sites in our study.

Another factor may be marketing of formula milk which has been shown to affect the breastfeeding behaviors of mothers [7,10]. Mothers are given the impression that formula milk is as good as, or better than, breast milk [10]. Marketing of formula milk may be more aggressive in urban areas than in rural areas in Vietnam. Economic conditions are also better in the urban area. Financial constraints may to a higher extent prevent women from buying formula milk in the rural areas [7].

The associations between household economic condition and duration of exclusive breastfeeding found in this study might partly explain the differences of breastfeeding practices of infants between the two settings.

Another factor behind the differences of breastfeeding practices between the two sites may be education. An earlier study in rural area of Vietnam showed that non-exclusive breastfeeding women had less education than exclusively breastfeeding women [7]. In the present study, the median duration of exclusive breastfeeding for the group of the highest level of education in the urban area was shortest. The Cox regression analysis pointed to a significant influence in the urban boy stratum. In the rural area, the result was the opposite: the median duration of exclusive breastfeeding for the group with the highest level of education was the longest. A problem for the interpretation of these results is that the statistical significance is very much dependent on numbers of observations. For education, the distributions are radically different between the urban and rural areas. Two reasonable hypotheses could be that the higher educated women in the urban area, who are the majority, tend to give exclusive breastfeeding shorter than others, and that the rather few women with higher education in the rural area for unclear reasons, breastfeed exclusively longer than rural women with low education.

The duration of exclusive breastfeeding by mothers with three antenatal care visits or more was shorter than the duration of exclusive breastfeeding of mothers with less than three visits. The result may indicate that many antenatal visits did not necessarily give mothers more information about breast feeding. A study in Vietnam found that lack of information and misunderstanding of mothers about breastfeeding were barriers to exclusive breastfeeding [12].

The results of this article show that exclusive breastfeeding decreased rapidly with increasing child age and was uncommon at six months of age at both sites. Similar results have been seen in China and in other studies in Vietnam. The percentage of children with exclusive breastfeeding dropped from 83.6% at the age of one week to zero at week 24 in a previous study in rural Vietnam [7]. The percentages of infants less than 6 months of age, who were exclusively breastfed, was 19.6% in Vietnam in 2010 [8]. In China, exclusive breastfeeding at six months of age dropped to 0.2% in an urban area and to 7.2% in a rural area [14]. The most important reason was that the mothers had to return to work [7,11,27]. Vietnamese mothers have a legal right to four months maternity leave [13], which is insufficient in relation to the WHO recommendation about six months of exclusive breastfeeding. Mothers commonly see formular milk as the best choice for the child when they return to work [10]. Another reason for giving complementary food was discussed in China, suggesting there is a belief that it will improve weight gain and lead to healthier babies [14]. A Chinese tradition is also that friends and relatives come to visit the mother and child after delivery. The most popular gift is infant milk formula. This is one reason for the extensive use of early formula milk in China [15].

The last major finding of this study is that most infants in both areas are reported to be given any breastfeeding for the first 12 months. Towards the end of the first year, breastfeeding is most rapidly declining in the urban area. Similar results were seen in Tanzania. Mean duration of breastfeeding was 27 ± 5 months in a rural compared to 24 ± 5 months in an urban area [16]. The opposite results were found in the USA: the prevalence of breastfeeding was significantly lower in a rural area than in an urban area [2]. Results from National Institute of Nutrition in Vietnam showed that 77% of infants in 2009–2010 continued breastfeeding at 12 months of age and that 22% continued breastfeeding at 24 months of age [8]. In Tanzania, reasons to stop breastfeeding early were that the child was considered old enough, mothers started a new pregnancy, the child refused the breastfeeding, infant or maternal illness or that mothers did not want to continue breastfeeding [16]. In Vietnam, some common reasons to stop breastfeeding have been found to be perceived insufficient breast milk supply, mothers return to work, that infants stopped taking breast milk and the mother’s health condition [5]. Even though the educational level of mothers was higher in the urban area, we found that any breastfeeding was lower in the urban compared to the rural area and also lower than data from National Institute of Nutrition in Vietnam [8]. One important reason behind that finding could be that urban mothers returned to work early.

Boys are considered more important than girls in Vietnam [28], but the present study found that boys were rather given less breastfeeding than girls in both sites at all ages. A reason may be that formula feeding is considered better than breastfeeding and hence preferred for the boys.

One limitation of the present study is the restriction to one year of follow-up. A second limitation of the study is that it is based on interview data from the mothers. Even if the interviewers were carefully trained to ask for data e.g. on additional foods from the whole period after birth or last interview, respectively, recall bias could not be excluded.

Moreover, it could not be excluded that hospital staff may have given early formula meals without the knowledge of the mother, particularly to babies born with Caesarean section. However, the present study shares these methodological problems with most other published breast feeding studies. Consequently, results from this study allows for comparison to data from other studies.

In conclusion, exclusive breastfeeding is still low in rural and more common in urban Vietnam. Intervention programs to promote breastfeeding are necessary particularly regarding early initiation and prolonged breastfeeding. To improve the latter a prolonged period of maternity leave to at least until the infant is six month old would be desirable.

## Conclusion

Initiation of breastfeeding during the first hour after birth was more common in the urban area than in the rural. Exclusive breastfeeding during the first months of life and breastfeeding in general was more frequent in the rural area than in the urban. Exclusive breastfeeding at six months of age of infants was uncommon in both areas.

The results suggest that the different breastfeeding practices between the two sites are not only due to the geographic location as such but rather to conditions associated with living in urban or rural settings.

To reduce the impact of such factors we propose that intervention programs with the aim of increasing the mother’s knowledge about breastfeeding, particularly rural mothers, should be implemented. A minimum of six months maternity leave would probably be an important measure to prolong the duration of exclusive breastfeeding. Limitation of advertisement and marketing of formula milk should be imposed.

## Competing interests

The authors declare that our findings have not been influenced by our personal or financial relationships with other persons or other organizations.

## Authors’ contributions

HNT led and supervised the fieldwork and data management. She also drafted and completed this paper. BE assisted in the research design as well as in the statistical analyses, interpretation of results and revising the manuscript. HA assisted particularly with paediatric expertise and was together with MP, GB, TTK, CNTK and NTL involved in the design of the study, supervised the study and revised the manuscript. All authors have read and approved the final manuscript.

## Pre-publication history

The pre-publication history for this paper can be accessed here:

http://www.biomedcentral.com/1471-2458/12/964/prepub
